# Effectiveness of Cognitive Behavioral Therapy in Reducing Suicidal Ideation and Influential Factors in Patients With Major Depressive Disorder: A Systematic Review and Meta‐Analysis

**DOI:** 10.1002/pchj.70034

**Published:** 2025-07-03

**Authors:** Mengzhen Zhao, Peng Wang

**Affiliations:** ^1^ School of Sociology Beijing Normal University Beijing China; ^2^ Department of Language, Literature and Communication, Faculty of Social Sciences and Humanities Vrije Universiteit Amsterdam Amsterdam the Netherlands; ^3^ Department of Psychology, Education, and Child Studies, Erasmus School of Social and Behavioural Sciences Erasmus University Rotterdam Rotterdam the Netherlands

**Keywords:** cognitive behavioral therapy, depression, suicidal ideation

## Abstract

To evaluate the effectiveness of cognitive behavioral therapy (CBT) interventions in reducing suicidal ideation among patients with depression, and to identify factors that moderate treatment outcomes, a systematic review and meta‐analysis was conducted searching seven major biomedical databases (CNKI, China Wanfang Database, Cochrane Library, PubMed, Web of Science, Embase, and Sinomed) from inception to November 8, 2023. Randomized controlled trials examining CBT interventions for suicidal ideation in depressed patients were included. Fourteen studies (1256 patients) met inclusion criteria. CBT demonstrated a moderate effect in reducing suicidal ideation compared to control conditions (Hedges' *g* = −0.47 (95% CI [−0.73, −0.22]), *p <* 0.01). Significant moderators included intervention mode (*Q* = 8.33, *p <* 0.05), suicidal ideation measure (*Q* = 17.98, *p <* 0.001), intervention duration (*Q* = 9.55, *p <* 0.05), and CBT follow‐up interval (*Q* = 6.66, *p <* 0.05). Age, intervention cycles, frequency, and intervention form did not significantly moderate outcomes. No publication bias was detected, and the overall quality of evidence was moderate. CBT is effective in reducing suicidal ideation among patients with depression. Factors associated with greater effectiveness include combining CBT with drug, targeting older adults, using specific suicidal ideation measures (e.g., C‐SSRS, SIOSS), interventions lasting 0–12 weeks with more than once a week, more than an hour per session, and short‐term follow‐up. Group therapy formats may also enhance outcomes. Future randomized trials should further examine these factors and assess impacts on suicidal behaviors in addition to ideation.

## 1 Introduction

Suicide remains a critical global health concern, with profound impacts on individuals, families, and communities worldwide. According to the World Health Organization's latest data, approximately 703,000 people die by suicide annually, with an estimated 20 times more individuals attempting suicide. This translates to one death every 40 s, underscoring the urgency of addressing this public health crisis (World Health Organization 2021). The risk is particularly elevated among those with major depressive disorder (MDD), where the suicide rate is approximately 20 times higher than in the general population (Omary 2021). Suicidal ideation and suicidal attempts are two critical indicators of suicide risk, with the former encompassing a spectrum of thoughts ranging from fleeting considerations to intense preoccupations with death (Harmer et al. 2020), and the latter involving non‐fatal, self‐directed, potentially injurious behavior carried out with the intent to die, regardless of resulting injury (Abdollahi and Talib 2015). The substantial overlap between symptoms of suicidal ideation and depression creates a complex interplay that heightens vulnerability to suicidal behavior, as both are strongly associated with depressive disorders. Research has consistently demonstrated a graded relationship between depression severity and suicidal risk (Naghavi 2019). Individuals experiencing depression are not only more likely to develop suicidal ideation but also to progress from ideation to actual suicide attempts (Ribeiro et al. 2018). This progression highlights the critical importance of early intervention and comprehensive mental health support for those suffering from depressive disorders.

Cognitive Behavioral Therapy (CBT) is widely recognized as the most extensively studied psychotherapy for depression, demonstrating efficacy in reducing depressive symptoms and preventing relapse (Hollon and Ponniah 2010). CBT targets distorted cognitions, maladaptive beliefs, and information‐processing biases—factors emphasized as central mechanisms in psychological models of depression, such as Beck's Cognitive Model (Beck and Haigh 2014) and Ellis's ABC Theory (Ellis 1991). By reshaping negative thought patterns and behaviors through techniques like increasing exposure to natural reinforcements and examining automatic negative thoughts, CBT effectively addresses cognitive distortions that serve as precursors to depressive disorders (Fortune and Goodie 2012). However, while distorted cognition is highlighted as a shared cognitive mechanism linking depression and suicidal ideation (Beck and Bredemeier 2016), prominent suicide models—including the Three‐Step Theory (Klonsky and May 2015), Psychological Pain Theory (Melzack 1993), and Interpersonal Theory of Suicide (Van Orden et al. 2010)—do not identify cognitive distortion as a critical proximal factor. This discrepancy underscores potential limitations in conventional understandings of suicide within existing literature, suggesting that caution is warranted when generalizing cognitive mechanisms from depression‐focused therapies like CBT to explanations of suicidal behaviors.

The relationship between CBT, depression, and suicidal ideation involves several key considerations. Research indicates that empirically supported treatments for depression may not always effectively address suicidal ideation, and the presence of suicidal ideation can sometimes limit the effectiveness of CBT for depression. For example, studies highlight that suicidal ideation may not serve as a reliable diagnostic indicator for depressive disorders (Pompili 2019). Individuals experiencing suicidal ideation often exhibit attentional fixation, which can interfere with rational information processing and make it difficult to disengage from suicide‐related thoughts (Wenzel et al. 2009). Furthermore, active suicidal ideation or behavior has been associated with poorer treatment outcomes and lower remission rates in patients with major depressive disorder (MDD) (Lopez‐Castroman et al. 2016).

Evidence suggests that CBT may have limited impact on reducing suicidal beliefs in depressed individuals. A study on CBT group psychotherapy for depressed adolescents found no significant advantage over routine care in addressing suicidal ideation (Wood et al. 2001). Similarly, two randomized controlled trials (RCTs) involving at‐risk adolescents reported that CBT was no more effective than usual care in reducing suicidal ideation or preventing the recurrence of suicidal behavior (Harrington et al. 1998; Rotheram‐Borus et al. 2000). These findings support the notion that suicidal ideation and depression may sometimes operate as distinct constructs due to differing underlying mechanisms (Bredemeier and Miller 2015). However, this view does not fully account for the frequent co‐occurrence of suicidal ideation and mental disorders, particularly depression (Chesney et al. 2014).

A transdiagnostic approach offers a broader perspective by identifying shared cognitive processes across multiple disorders. According to this framework, cognitive dysfunction may play a central role in both psychopathological disorders and suicidal ideation (Bredemeier and Miller 2015). Within the cognitive model of CBT, dysfunctional thought patterns are considered a key factor contributing to mental illnesses (Wang et al. 2024). In MDD specifically, high levels of suicidal ideation have been linked to deficits in cognitive control—the ability to regulate thoughts and behaviors to achieve goals and adapt to changing circumstances (Braver 2012). These cognitive impairments may increase MDD patients' sensitivity towards their surroundings, potentially rendering them more susceptible than healthy individuals to interpret neutral events as “setbacks” or “neglect” (Oquendo et al. 2024). This heightened sensitivity can lead to the identification of triggers for suicidal thoughts and behaviors (Rudd et al. 2001). For addressing suicidal ideation among depressed patients, CBT employs a multifaceted approach. It discerns recent thoughts underlying suicide attempts, resolves maladaptive cognitive and behavioral strategies, and fosters adaptive coping abilities in response to stress. This comprehensive strategy aims to empower individuals to decrease both suicidal ideation and depressive symptoms. Supporting this approach, a meta‐analysis of 28 controlled studies (Tarrier et al. 2008) found that CBT significantly reduced suicide risk and feelings of hopelessness. While this integrated perspective highlights the potential of CBT in addressing both depression and suicidal ideation through shared cognitive mechanisms, recent findings have revealed a more complex picture. The efficacy of CBT in reducing suicidal ideation among depressed individuals remains a subject of debate in the scientific community. Notably, there is a conspicuous absence of recent meta‐analyses specifically examining the effect of CBT on suicidal ideation in depressed patients, along with potential modifying factors and sources of heterogeneity that may influence treatment outcomes. To address this critical gap in the literature, our meta‐analysis aims to comprehensively evaluate the effectiveness of CBT in reducing suicidal ideation among individuals with depression. We extend our investigation beyond mere efficacy assessment to explore the nuanced factors that may modulate treatment outcomes. Specifically, we examine the following factors:

1. The overall effectiveness of CBT in reducing suicidal ideation across diverse depressed populations, including youth and older adults.
2. The potential moderating effects of intervention parameters such as treatment duration, frequency, and session length.
3. The impact of different measurement tools used to assess suicidal ideation.

By conducting this comprehensive analysis, we aim to provide clinicians and researchers with robust, evidence‐based insights into the efficacy of CBT for addressing suicidal ideation in depressed patients. Our findings will not only contribute to the ongoing discourse on best practices for treating comorbid depression and suicidal ideation but also inform future research directions and clinical interventions in this critical area of mental health care.

## 2 Methods

This systematic review and meta‐analysis was reported following the Preferred Reporting Items for Systematic Reviews and Meta‐analyses (PRISMA) reporting guideline (Liberati et al. 2009). We have registered on the PROSPERO International Systematic Evaluation Registration Platform with ID CRD42024531343.

Table 1 displays the inclusion/exclusion criteria used in our review, formulated under the Population Intervention Comparator Outcome Study (PICOS) design model. We combined search terms related to depression, suicidal ideation, CBT, and RCTs using Boolean operators (see Appendix S1 for full search strategy). Two independent reviewers (C.c. and G.n.) screened seven electronic literature databases for RCTs examining the effectiveness of CBT in treating suicidal ideation among patients with depression. The databases searched were CNKI, China Wanfang Database, Cochrane Library, PubMed, Web of Science, Embase, and Sinomed. The search covered the period from database inception until November 8, 2023. We used both medical subject headings (MeSH) and text word terms, including: (Depression OR “depressive symptoms” OR “emotional depression” OR “Depressive”) AND (“cognitive behavioral therapy” OR “cbt” OR Cognitive OR behav* OR “cognitive psychotherap*”) AND (suicide OR “suicidal behavior” OR “suicidal ideation”) AND (“Randomized controlled trial” OR Randomized OR controlled OR Trial).

**TABLE 1:** The inclusion and exclusion criteria presented in a PICOS framework.

PICOS	Inclusion criteria	Exclusion criteria
Population	Patients diagnosed with major depressive disorder using standardized criteria (DSM‐III, DSM‐III‐R, DSM‐IV‐TR, DSM‐5, or ICD‐10) Participants meeting depression thresholds on validated symptom questionnaires	Individuals without clinical depression Participants with mild or subclinical depressive symptoms
Invention	Patients in the intervention group received CBT. Based on previous definitions of CBT in the literature, CBT is explicitly described as CBT, including one or more of the following components: cognitive reconstruction, behavioral activation, in vivo or sensory exposure, problem‐solving, coping skills training, and behavioral experimentation. Cognitive reconstruction. The study can be included as long as one or more of these components are included. So the types of interventions included in this study are dominated by classical CBT techniques.	Patients in the intervention group did not receive CBT (Dialectical behavior therapy and mindful behavior therapy).
Comparison	Participants who received routine care, medication, supportive counseling, or no intervention.	Patients who received other psychotherapy treatment
Outcome	Suicidal ideation of patients, which were measured by various psychological scales.	Outcome indicators did not involve suicidal ideation
Study design	Studies are randomized controlled trial. A quantitative analysis of outcome is included.	No quantitative analysis of outcome is included. Studies which are not randomized controlled trials. Articles written in languages other than English. Articles which have not been peer‐reviewed (i.e., gray literature).

Abbreviation: PICOS, Population Intervention Comparator Outcome Study design.

The two reviewers independently screened the literature according to the predefined inclusion and exclusion criteria, using a double‐blind approach by EndNote. The screening process involved two stages. First, reviewers read titles and abstracts to preliminarily exclude irrelevant articles. For potentially eligible studies, reviewers then downloaded and carefully read the full text for detailed screening. After completing the screening process, the two researchers compared their selected literature. In cases of disagreement, a third reviewer was consulted to jointly discuss whether to include the study in question.

### 2.1 Data Extraction

Two reviewers separately performed data extraction and compared their extracted data, focusing on the effectiveness of CBT versus control therapy in decreasing suicidal thoughts. Extracted data pertained to the impact of CBT and control groups. Quantitative data from key outcome assessments were collected over all available intervals. Alongside numerical outcomes, data points such as literature information (author, year of publication, country), study methodology, form, location, sample size, participant demographics (age, number), interventions offered, total participants, analyzed participants, evaluation time, and main outcome indicators were included.

### 2.2 Quality Assessment

We employed the Physiotherapy Evidence Database (PEDro) scale (Verhagen et al. 1998) to evaluate the quality of the included literature. This scale consists of 11 items, with each item scored as either “Yes” (1 point) or “No” (0 point). The first item serves as an external eligibility indicator and is not included in the final score. Therefore, the maximum possible score is 10 points. The methodological quality of included studies was classified into three tiers based on cumulative assessment scores: studies achieving total scores ≥ 6 were designated as high methodological quality (with scores 6–8 categorized as good and 9–10 as excellent), those scoring ≤ 5 were classified as low methodological quality (scores 4–5 representing fair quality), while studies attaining scores below 4 were categorized as poor quality (Amer‐Cuenca et al. 2023). Two independent researchers conducted the literature quality scoring. In cases of disagreement, a third reviewer was consulted, and discussions were held until consensus was reached. We utilized GRADEpro software to assess the quality of outcome evidence. This assessment included five evaluation criteria: limitations, inconsistency, indirectness, imprecision, and publication bias. Each criterion was rated as none (no downgrade), serious (downgrade by 1 level), or very serious (downgrade by 2 levels). The evidence was classified into four quality levels (very low, low, moderate, and high) reflecting the degree of confidence in the estimated effects (Chen et al. 2017), as detailed in Table 2.

**TABLE 2:** The definition of quality level.

Quality level	Definition
High	High confidence that the true effect closely matches the estimated effect
Moderate	Moderate confidence—true effect is likely close to estimate but may differ substantially
Low	Limited confidence—true effect could differ substantially from estimate
Very low	Very little confidence—true effect likely differs substantially from estimate

### 2.3 Quantitative Data Synthesis

We used Stata 17.0 software to conduct data synthesis, heterogeneity testing, bias analysis, and subgroup analysis. Hedges's *g* and its 95% confidence interval were used as indicators of effect size for suicidal ideation outcomes. Following established guidelines (Cohen 1992), we interpret effects as small (0.2), medium (0.5), or large (0.8). *p*‐values and *I* ^2^ statistics were employed to test for heterogeneity among the studies. A *p*‐value greater than 0.10 indicated no heterogeneity, while a *p*‐value less than 0.10 indicated heterogeneity. Higgins' *I* ^2^ statistic was interpreted as follows: high heterogeneity (75%), medium heterogeneity (50%), and low heterogeneity (25%) (Higgins et al. 2003). According to Cochrane's recommendations, heterogeneity should not exceed 40% to be considered acceptable. In such cases, a fixed‐effect model can be used for analysis. If heterogeneity is greater than 40%, a random‐effects model is employed, and subgroup analysis is conducted to identify potential sources of heterogeneity. We used the Egger test in Stata 17.0 software to perform bias analysis, assessing the potential for publication bias.

## 3 Results

**FIGURE 1:**
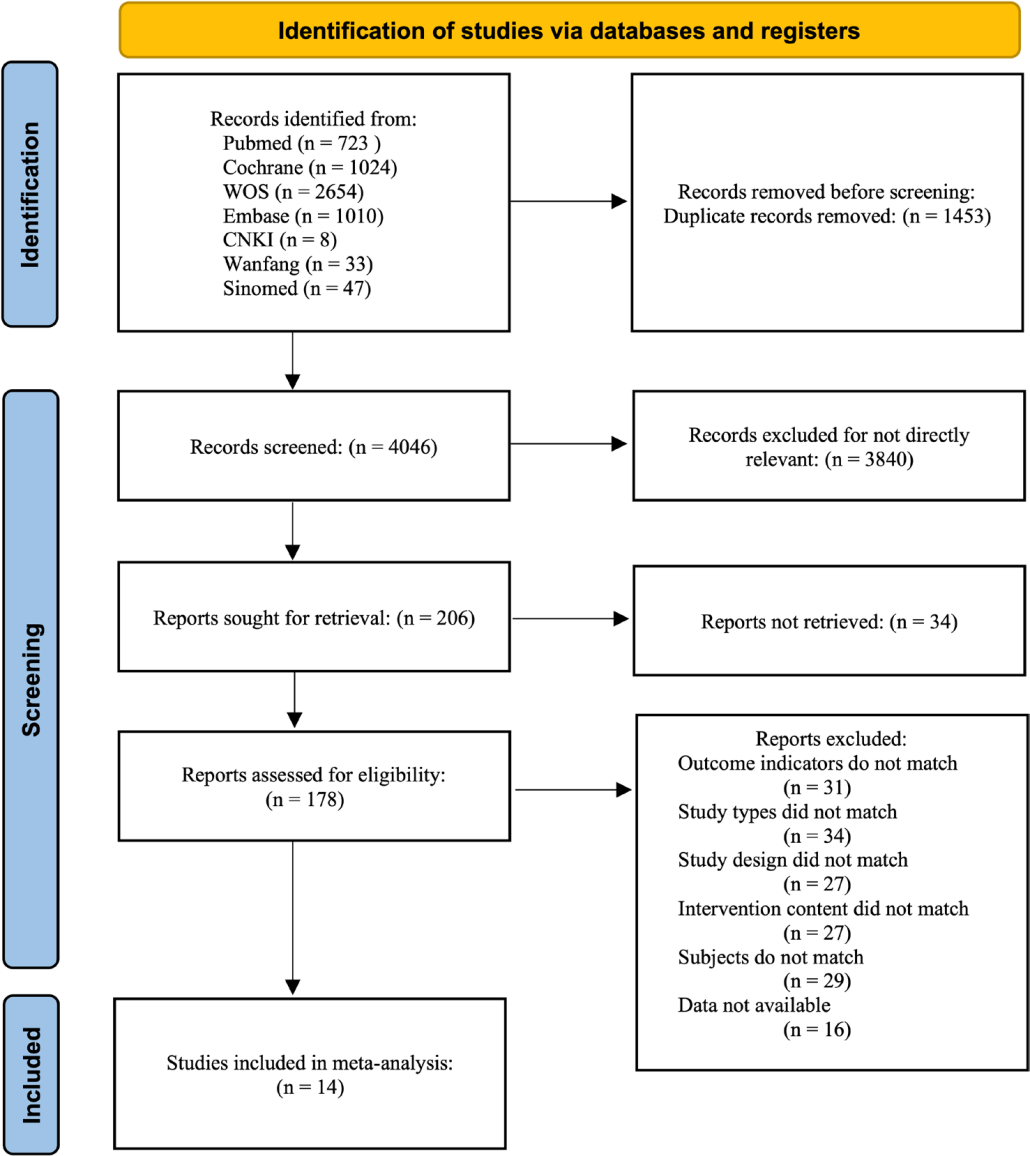
PRISMA flow diagram.

Our systematic search strategy initially identified 5499 studies, which was reduced to 4046 unique articles after removing 1453 duplicates (Figure 1). Following title and abstract screening, we excluded 3840 articles, leaving 206 for potential full‐text review. We successfully obtained 6 of 34 initially inaccessible full‐text articles by contacting the corresponding authors. Subsequently, 178 studies underwent comprehensive full‐text assessment for eligibility. After independent evaluation, 14 articles met the inclusion criteria and were incorporated into the systematic review. The primary reasons for exclusion during full‐text review were: mismatched outcome indicators (*n* = 31), inappropriate study design (*n* = 34), unsuitable intervention content (*n* = 27), non‐RCTs (*n* = 27), ineligible study population (*n* = 29), and inability to extract data (*n* = 16). Fourteen studies (Brenner et al. 2018; Jacobs et al. 2009; LaCroix et al. 2018; March et al. 2004; Melvin et al. 2006; Pigeon et al. 2019; Pratt et al. 2015; Rudd et al. 2015; Sayal et al. 2019; Sinniah et al. 2017; Slee et al. 2008; Wei et al. 2021; Li et al. 2017; Wu et al. 2022) involving 1256 subjects were included, with 623 samples in the experimental group and 633 in the control group. The research subjects ranged in age from 13 to 74 years and included youth (Jacobs et al. 2009; LaCroix et al. 2018; March et al. 2004; Melvin et al. 2006; Pratt et al. 2015; Rudd et al. 2015; Sayal et al. 2019; Slee et al. 2008; Wei et al. 2021; Wu et al. 2022), middle‐aged, and elderly groups (Brenner et al. 2018; Pigeon et al. 2019; Sinniah et al. 2017; Li et al. 2017). The studies originated from four continents: Asia (Sinniah et al. 2017; Wei et al. 2021; Li et al. 2017; Wu et al. 2022), America (Brenner et al. 2018; Jacobs et al. 2009; LaCroix et al. 2018; March et al. 2004; Pigeon et al. 2019; Rudd et al. 2015), Europe (Pratt et al. 2015; Sayal et al. 2019; Slee et al. 2008), and Australia (Melvin et al. 2006).

Table 3 shows 14 qualified studies reported on CBT interventions and suicide ideation variables. The quality of the studies was assessed using the PEDro scale, with an average score of 6.5 points. The scores ranged from 5 points (Sayal et al. 2019; Wei et al. 2021; Li et al. 2017; Wu et al. 2022), 6 points (LaCroix et al. 2018; Melvin et al. 2006; Sinniah et al. 2017), 7 points (Jacobs et al. 2009; March et al. 2004; Pigeon et al. 2019), to 8 points (Brenner et al. 2018; Pratt et al. 2015; Rudd et al. 2015; Slee et al. 2008). The studies provided comprehensive details on CBT interventions, including intervention content, form, cycle, duration, and frequency. Specifically, the interventions included both individual and group formats, with cycles ranging from 0 to 24 weeks, and frequencies from 0.5 to 6 times per week. The intervention sites varied and included hospitals, prisons, mental health institutes, and homes.

**TABLE 3:** Study characteristics.

Study, year, country	Study quality	Depression diagnostic criteria (cut‐off points)	Location	Sample	Age	Intervention content		Intervention features	Data collection	Outcome measures
				E/C	E/C (MA)	E	C	Cycle, times, duration, frequency		
Slee et al. (2008), Netherlands	8	BDI‐II (≥ 17)	Hospital	40/42	23.9/25.4	BCBT	Medication	24 weeks, 12 times, 2 h each time, once a week	Baseline, 3‐month, 6‐month and 9‐month follow‐up	SCS
Brenner et al. (2018), USA	7	BDI‐II (≥ 17)	Community hospitals	15/20	47.7/54.6	GCBT	Waitlist	10 weeks, 10 times, 2 h each time, once a week	Baseline, 3‐month, 6‐month follow‐up	BSS
LaCroix et al. (2018), USA	6	BDI‐II (≥ 17)	Hospital	18/18	28.9/33.0	PACT	Routine care	3 days, 6 times, 60–90 min each time, 6 times a week	Baseline, 1‐month, 2‐month and 3‐month follow‐up	BSS
Rudd et al. (2015), USA	7	BDI‐II (≥ 17)	Community hospitals	76/76	27.18/27.6	BCBT	Routine care	6–12 weeks, 12 times, 90 min for the first time, then 60 min each time, once a week combined with every 2 week	Baseline, 3‐month, 6‐month 12‐month 18‐month and 24‐month follow‐up	BSS
Pigeon et al. (2019), USA	6	PHQ‐9 (> 15)	Community hospitals	24/26	52.79/56.8	BCBT	Routine care	5 weeks, 4 times, 30 min each time, once a week combined with once 2 week	Baseline, posttreatment, 6‐month follow‐up	CSSRS
Pratt et al. (2015), UK	8	BDI‐II (≥ 17)	Prison	31/31	38.5/32.0	CBSP	Routine care	More than 20 weeks, 20 times, 1 h each time, once a week combined with every 2 week	Baseline, 4‐month, 6‐month follow‐up	BSS
Sayal et al. (2019), UK	6	BDI‐II (≥ 17)	Home	11/11	21.0/20.0	PSCBT	Routine care	5–12 weeks, 10–12 times, 1 h each time, once a week combined with every 2 week	Baseline, 3‐month, 6‐month, 9‐month and 12‐month follow‐up	CSSRS
Sinniah et al. (2017), Malaysia	6	BDI‐II (≥ 17)	Hospital	33/36	43.1 (WSA)	ICBT	Routine care	8 weeks, 16 times, 2 h each time, twice a week	Baseline, 4‐week, 8‐week, 3‐month and 6‐month follow‐up	BSS
Jacobs et al. (2009), USA	8	DSM‐5	Mental health Institute	111/107	14.6 (WSA)	CCBT	Medication	12 weeks, 16 times, 2 h each time, NR	Baseline, 6‐week, 12‐week	SIQ‐Jr
March et al. (2004), USA	7	DSM‐5	Hospital	111/107	14.6 (WSA)	CBT	Medication	12 weeks, 15 times, 50–60 min each time, NR	Baseline, 6‐week, 12‐week	SIQ‐Jr
Melvin et al. (2006), Australian	8	DSM‐5	Community hospitals	22/25	15.3 (WSA)	CBT	Medication	12 weeks, 12 times, 50 min each time, once a week	Baseline, posttreatment	SCS
Li et al. (2017), China	5	ICD‐10	Hospital	45/50	68.9/68.3	CBT	Medication	8 weeks, 16 times, 1 h each time, twice a week	Baseline, posttreatment	SIOSS
Wu et al. (2022), China	5	Standardized diagnostic criteria for depression	Hospital	55/55	14.9/14.3	CBT	Medication	8 weeks, 18/19, NR, twice a week	Baseline, posttreatment	QSA
Wei et al. 2021, China	5	HAMD (≥ 20)	Hospital	31/29	33.4 (WSA)	GCBT	Medication	6 weeks, 12 times, 1.5–2 h each time, twice a week	Baseline, posttreatment	SIOSS

Abbreviations: BCBT, Brief Cognitive Behavioral Therapy; BDI, Beck Depression Inventory; BSSI, Beck Scale for Suicide Ideation; C, comparator group; CBSP, Cognitive Behavioral Suicide Prevention; C‐SSRS, Columbia‐suicide severity rating scale; E, experimental group; GCBT, Group Cognitive Behavioral Therapy; HAMD, Hamilton Depression Scale; ICBT, Individual Cognitive Behavioral Therapy; ICD‐10, Tenth Revision of the International Classification of Diseases; MA, mean age; NR, not reported; PACT, Post‐Admission Cognitive Therapy; PSCBT, Problem Solving Cognitive Behavioral Therapy; QSA, Suicide attitude questionnaire; SIOSS, Reduction in self‐rating idea of suicide scale; SIQ‐Jr, Suicide ideation questionnaire‐junior; SCS, Suicide Cognition Scale; WSA, whole sample mean age.

**FIGURE 2:**
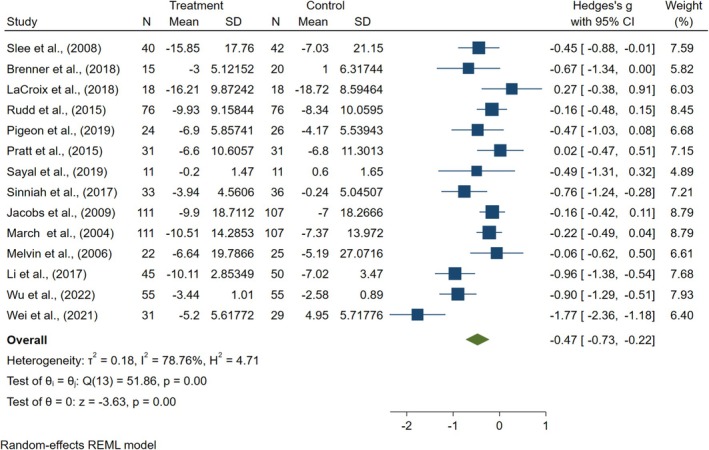
Forest plot for cognitive behavioral therapy on reducing suicidal ideation in the pooled meta‐analysis.

**FIGURE 3:**
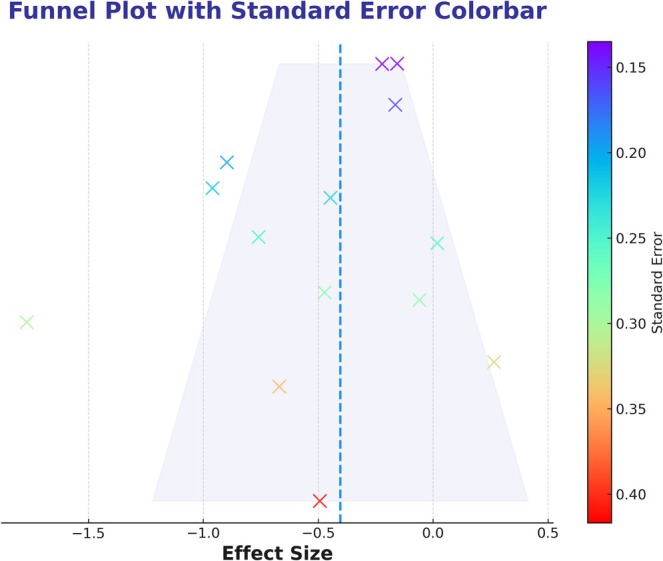
Funnel plot for cognitive behavioral therapy on reducing suicidal ideation in the pooled meta‐analysis.

**FIGURE 4:**
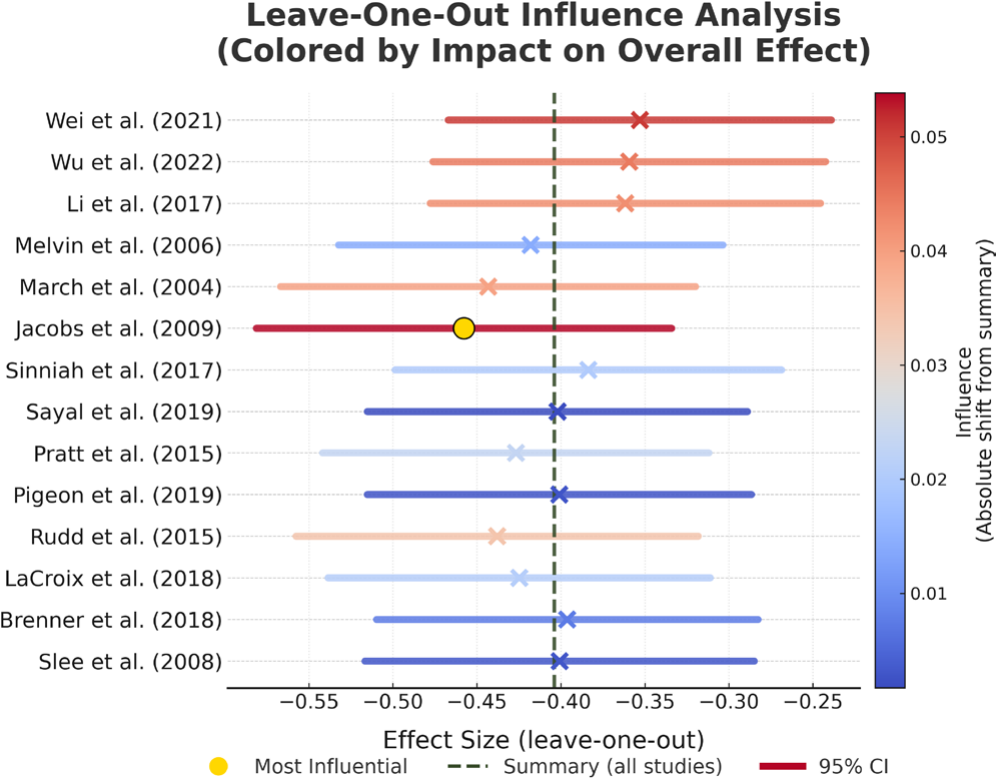
Sensitivity analysis for cognitive behavioral therapy on reducing suicidal ideation in the pooled meta‐analysis.

The meta‐analysis revealed a significant improvement in the main outcome for CBT compared to control treatments. The mean effect size was *g* = −0.47 (95% CI [−0.73, −0.22], *p* < 0.012), as illustrated in Figure 2. However, substantial heterogeneity was observed (*I* ^2^ = 78.76%, *p* < 0.050). The Egger test (*Z* = −0.59, *p* = 0.562) indicated no significant publication bias. However, from the perspective of the tunnel plot, the presence of three studies falling outside the 95% confidence boundaries of the funnel plot suggests potential asymmetry attributable to inter‐study heterogeneity, as shown in Figure 3. Sensitivity analyses, conducted by sequentially excluding individual trials (Figure 4), identified no studies as the primary source of heterogeneity, which suggests robust overall results. Using GRADEpro software, we evaluated the quality of evidence. Despite high heterogeneity, we conducted subgroup analysis to find that the identification of its source mitigated concerns and did not downgrade one level for inconsistency. The remaining four GRADE criteria were not downgraded, resulting in a high overall quality of evidence for the outcome indicators (Table 4).

**TABLE 4:** Evidence evaluation of effects of CBT on suicide ideation.

Quality assessment							No. of patients		Effect	Quality
No. of studies	Design	Risk of bias	Inconsistency	Indirectness	Imprecision	Other considerations	CBT	Not CBT	Absolute	
14	Randomized trials	No serious risk of bias	No serious inconsistency^a^	No serious indirectness	No serious imprecision	None	623	633	−0.47 (−0.73, −0.22)	High

^a^ Intervention content, measurement tool, intervention time, and CBT following‐up time all played a moderating role through subgroup analysis.

The subgroup analyses revealed significant moderating effects on the efficacy of CBT for suicidal ideation (Table 5). Intervention content also significantly moderated outcomes (*Q* = 8.33, *p* < 0.050), with CBT combined with drug showing a large effect (*g* = −0.99, 95% CI [−1.49, −0.49], *p* < 0.051), CBT alone with a small effect (*g* = −0.21, 95% CI [−0.38, −0.036]), while CBT with routine nursing did not yield significant results. The measurement instrument used also influenced the observed effects: the SIOSS showed the largest effect size (*g* = −1.34, 95% CI [−2.13, −0.55], *p* < 0.051), followed by C‐SSRS (*g* = −0.48, 95% CI [−0.94, −0.02], *p* < 0.051), while studies using BSSI, SIQ‐Jr did not show a significant effect. Intervention time emerged as a significant moderator (*Q* = 9.55, *p* < 0.001), with more time (> 1 h per) showing a larger effect size (*g* = −0.58, 95% CI [−1.08, −0.09], *p* < 0.056) compared to less intervention time. The timeliness of the intervention effect also played a crucial role (*Q* = 6.66, *p* = 0.040), with short‐term effects immediately post‐intervention demonstrating the most substantial impact (*g* = −0.89, 95% CI [−1.38, −0.39], *p* < 0.050). Our analysis found that age did not significantly moderate the efficacy of CBT for reducing suicidal ideation, as indicated by a non‐significant Q statistic (*Q* = 3.07, *p* > 0.052). Similarly, intervention cycle (*Q* = 1.12, *p* = 0.291), frequency (*Q* = 4.13, *p* = 0.136), and format (group vs. individual therapy, *Q* = 2.37, *p* = 0.122) did not significantly moderate treatment outcomes. These findings suggest that while age does not significantly moderate CBT efficacy overall, certain age groups may exhibit different responses to treatment. The most robust effects were observed when CBT was combined with medication, assessed immediately post‐intervention, and when using specific measurement tools like the SIOSS.

**TABLE 5:** Moderator analyses of effects of CBT on suicide ideation.

Subgroup analysis	Test of moderators	*N*	Hedge's, 95% CI	*p*
Age	*Q* = 3.07, *p* = 0.08			
Youth group (≤ 40 years old)		10	−0.38 [−0.72, −0.05]*	0.032
Middle‐aged and elderly (> 40 years old)		4	−0.76 [−1.01, −0.50]***	0.001
Intervention content	*Q* = 8.33, *p* = 0.02			
CBT alone		4	−0.21 [−0.38, −0.04]*	0.022
CBT with routine care		4	−0.26 [−0.55, 0.03]	0.070
CBT with drug		6	−0.99 [−1.49, −0.49]***	0.001
Outcome measurement	*Q* = 17.98, *p* = 0.00			
BSSI		5	−0.26 [−0.62, 0.10]	0.151
CSSRS		2	−0.48 [−0.94, −0.02]*	0.041
QSA		1	−0.90 [−1.29, −0.51]***	0.006
SIOSS		2	−1.34 [−2.13, −0.55]***	0.001
SIQ‐Jr		3	−0.18 [−0.35, 0.00]	0.050
SCS		1	−0.45 [−0.88, −0.01]*	0.042
Intervention cycles	*Q* = 1.12, *p* = 0.291			
0–12 weeks		12	−0.52 [−0.81, −0.23]*	0.026
12–24 weeks		2	−0.23 [−0.68, 0.23]	0.322
Intervention time	*Q* = 9.55, *p* = 0.01			
Less than 1 h		6	−0.25 [−0.43, −0.07]*	0.011
More than 1 h		7	−0.59 [−1.07, −0.12]*	0.021
NR		1	−0.90 [−1.29, −0.51]***	0.009
Intervention frequency	*Q* = 4.13, *p* = 0.13			
Less than once a week		7	−0.27 [−0.45, −0.08]*	0.018
More than once a week		5	−0.83 [−1.43, −0.24]*	0.015
NR		2	−0.19 [−0.38, 0.00]	0.005
Intervention form	*Q* = 2.37, *p* = 0.12			
Individual treatment		12	−0.37 [−0.58, −0.16]***	0.001
Group treatment		2	−1.23 [−2.31, −0.15]*	0.036
Intervention timeliness	*Q* = 6.66, *p* = 0.04	12		
Posttreatment		5	−0.89 [−1.38, −0.39]***	0.001
Less than 3‐month follow‐up		6	−0.20 [−0.35, −0.06]*	0.011
More than 3‐month follow‐up		3	−0.28 [−0.63, 0.07]	0.126

Abbreviations: CI, confidence intervals; N, number.

***p < 0.001; **p < 0.01; *p < 0.05.

## 4 Discussion

This systematic review and meta‐analysis demonstrates that CBT interventions are generally effective in reducing suicidal ideation compared to control treatments. Our analysis of 14 studies involving 1256 participants across four continents revealed several important findings. CBT appeared to be particularly effective when combined with pharmacotherapy, while CBT alone showed modest benefits. The effectiveness of interventions varied depending on several factors, including session duration, measurement tools used, and the timing of assessment, with immediate post‐intervention measurements showing the strongest positive effects. Although age did not significantly moderate overall outcomes, middle‐aged and elderly groups appeared to derive greater benefits than youth populations. These findings underscored the potential of CBT as a therapeutic approach for addressing suicidal ideation, while highlighting the importance of considering intervention characteristics and patient demographics when designing treatment programs. The high‐quality evidence supporting these conclusions, despite some heterogeneity across studies, strengthens confidence in CBT as an evidence‐based intervention for suicide prevention.

### 4.1 Potential Reasons Behind Efficiency of CBT in Reducing Suicidal Ideation

The efficiency of CBT interventions on suicidal ideation reduction may be realized through multiple complementary pathways. Our moderator analyses revealed that CBT combined with pharmacotherapy demonstrated the strongest effects, suggesting potential synergistic interactions between cognitive restructuring techniques and neurochemical modulation (Ray et al. 2020). This aligns with neuroimaging evidence indicating that CBT modulates prefrontal–limbic pathways critical for emotion regulation, with reduced activation in the amygdala and increased prefrontal cortical engagement (Månsson et al. 2016). The observed efficacy of longer intervention sessions (> 1 h) producing larger effect sizes supports the neuroplasticity hypothesis, whereby repeated therapeutic exposure facilitates structural and functional brain changes (Wager et al. 2008). Specifically, CBT appears to mitigate suicidal ideation by enhancing cognitive control capacities and improving emotion regulation strategies that are typically impaired in suicidal individuals (Nakao et al. 2021; Wang et al. 2025). The immediacy of treatment effects, with the strongest outcomes observed immediately post‐intervention, reflects CBT's direct targeting of maladaptive cognitive patterns through techniques such as cognitive restructuring and behavioral activation (Wang et al. 2024). Furthermore, the differential effectiveness across measurement tools suggests that CBT might influence distinct cognitive‐emotional dimensions of suicidality, with particularly robust effects on hopelessness and suicide‐specific cognitions as measured by specialized instruments (Kim et al. 2003).

However, it is important to note that not all studies have demonstrated consistent benefits of CBT, particularly among adolescents. Some trials have reported no significant advantage of CBT over control conditions in reducing suicidal ideation in youth populations (Harrington et al. 1998; Rotheram‐Borus et al. 2000). This divergence may be partly explained by developmental differences in cognitive flexibility and meta‐cognitive skills, which are still maturing during adolescence. Younger individuals may have more difficulty engaging with core CBT components such as cognitive restructuring, limiting the intervention's effectiveness in this group. Additionally, factors such as family environment, comorbidities, and varying levels of problem‐solving skills may further moderate outcomes among adolescents. These inconsistencies highlight the need for developmentally tailored CBT protocols and further research into mechanisms of change for different age groups.

Collectively, these findings indicated that while CBT's therapeutic efficacy in reducing suicidal ideation was supported by neurobiological and psychological mechanisms, its effectiveness might vary based on developmental stage and individual cognitive capacities. Future research should explore these moderating factors to optimize CBT interventions for diverse populations.

### 4.2 Moderating Effect of Intervention Parameters

The effectiveness of CBT in reducing suicidal ideation among patients with depression is influenced by several subgroup variables related to the intervention. These include the specific content of CBT, the duration of individual sessions, and the overall length of the treatment program. Interestingly, factors such as the patient's age, frequency of interventions, number of treatment cycles, and the form of CBT delivery did not significantly impact outcomes, addressing our second research question.

The content of the intervention significantly moderated the suicidal ideation in depressed patients undergoing CBT. Notably, the combination of CBT with pharmacological treatment proved more effective than CBT alone, aligning with findings from previous studies (Thase et al. 1997). While CBT alone focuses on altering negative cognitions and maladaptive behaviors to mitigate negative effects, it often overlooks the impact of biological, psychological, and environmental factors on suicidal ideation and behavior (Gournellis et al. 2018). In contrast, combining CBT with medication addresses a broader range of factors, thereby enhancing the overall effectiveness of the intervention.

The duration of CBT sessions also plays a moderating role in the effectiveness of the intervention, as verified by prior studies (Choi et al. 2016). Previous research observed that sessions lasting only 15–30 min in outpatient clinics did not significantly improve patient adherence to therapy (Petry 2005). The timeliness of CBT intervention significantly impacted effect. While the short‐term effects of CBT on reducing suicidal ideation are statistically significant and consistent with previous research (Thase et al. 1997), the long‐term effects are less clear. Some studies have found no significant long‐term reduction in suicidal ideation (Cuijpers et al. 2009), which also contradicts other research that followed patients for more than a year (Brown et al. 2005). Therefore, further evidence is needed to confirm the long‐term effectiveness of CBT in reducing suicidal ideation among depressed patients.

Routine care refers to a range of standard care services provided based on individual patient needs. In the studies reviewed, patients typically received usual care on the inpatient unit in addition to study‐related assessments. This routine care often included group psychotherapy, recreational therapy, art therapy, and medication management provided by nurses or medical staff (LaCroix et al. 2018). However, the specific components and intensity of routine care varied considerably across studies (Etzelmueller et al. 2020). Importantly, the providers delivering routine care were often non‐specialists without formal CBT training, such as psychologists lacking CBT education, registered nurses, general practitioners, and facilitators with only personal experience (Etzelmueller et al. 2020). This variability in provider background and training likely influenced the effectiveness of interventions, as studies suggest that routine care delivered by non‐specialists may not provide the same therapeutic benefit as care overseen by trained clinicians (Wang et al. 2024). The underexplored heterogeneity in routine care and provider expertise represents a limitation that could impact the comparability of outcomes across studies. Future research should more clearly define and standardize routine care protocols and report provider qualifications to better assess their influence on treatment effectiveness.

The frequency and cycle of CBT sessions showed no substantial impact on suicidal ideation outcomes among depressed patients. However, previous studies have shown that CBT treatment administered 1–2 times per week, with an intervention cycle of up to 12 weeks, tends to yield premium outcomes (Huang et al. 2015; Strachowski et al. 2008). Some researchers suggest that a higher ‘dose’ of CBT, meaning more frequent or longer‐duration treatments, may lead to greater overall efficacy (Beck and Dozois 2011). This divergence between our findings and prior research underscores the need for further exploration into the ideal frequency and duration of CBT interventions for effectively managing suicidal ideation in depressed patients.

The form of intervention (group vs. individual therapy) did not display a significant moderating role in CBT treatments for suicidal ideation in depressed patients. Some studies have shown that group therapy can be as effective as individual therapy (Kellett et al. 2007), which aligns with previous research findings (Tarrier et al. 2008). This equivalence may be attributed to several factors. For instance, suicidal ideation tends to be recurrent (Williams et al. 2006), suggesting that patients with depression have specific cognitive biases (Oquendo and Currier 2009). In this context, group therapy may exert a stronger social influence than individual therapy in modifying patients' suicidal ideation. However, it is important to note that in some cases, patients with severe depressive symptoms or high suicide risk might struggle to engage effectively in group therapy. In such situations, therapists have limited opportunities to address these patients' unique needs, highlighting the importance of individualized approaches in mental health treatment (Oei and Dingle 2008).

### 4.3 Measurement Effect

The various measurement tools used to assess suicidal thoughts moderated the outcomes, addressing our third research question. The largest effect size for suicidal ideation was found with the Suicidal Ideation and Behavior Scale (SIOSS), with Hedges's *g* = −1.34, followed by the Columbia‐Suicide Severity Rating Scale (C‐SSRS), with Hedges's *g* = −0.48. Both scales demonstrated significant moderation of suicidal thoughts. Compared to other scales, SIOSS, which includes four dimensions—optimism, sleep, despair, and covering‐up—showed high sensitivity and specificity in classifying suicidal behavior (Xia et al. 2002). However, it is important to note that SIOSS includes non‐traditional dimensions such as “optimism,” which may conflate suicidal ideation with broader psychopathology, potentially inflating its effect size relative to scales that focus more narrowly on suicidal thoughts and behaviors. This broader construct coverage could explain why SIOSS outperformed other scales like the BSSI. Similarly, the C‐SSRS is noted for its high sensitivity and specificity (Posner et al. 2011), possibly due to its comprehensive assessment of both the severity and intensity of suicidal ideation, but it may also capture a wider range of psychological distress. Thus, while both SIOSS and C‐SSRS demonstrated strong performance, their broader scope may partly account for the observed differences in effect sizes compared to more narrowly focused tools.

SIQ‐JR is used as a self‐report scale to identify youth with significant levels of suicidal ideation (Reynolds and Mazza 1999). However, its efficacy appears somewhat inadequate for this demographic, potentially due to gender biases. It has been more effective in predicting suicide attempts among female adolescents, while its predictive power is less clear for male adolescents, who are at elevated risk (King et al. 2014). BSSI is applicable to adults, but its suitability for youth remains uncertain (Beck et al. 1979).

The adequacy of indicators used to evaluate efficacy and the sensitivity of outcome indicators to changes in suicidal ideation are crucial in measuring suicidal thoughts. Given the range of suicidal ideation measurement tools and their differences in subjective perceptions across various populations and countries (Ghasemi et al. 2015), there are notable differences in how these tools measure suicidal ideation. Researchers must carefully select an appropriate scale for population screening due to the cross‐cultural nuances in suicidal ideation and behaviors.

### 4.4 Strengths and Limitations

Our study has several limitations that warrant consideration. Firstly, we were unable to examine multiple moderators simultaneously in a single model. This limitation prevents us from exploring potential interactions among variables such as intervention time, form, duration, frequency, and suicidal ideation measurement methods in influencing treatment effects. Unidentified interactions could potentially explain variances unaccounted for by our single‐variable moderator analyses. Consequently, our results should be interpreted cautiously, and future studies should aim to incorporate multiple moderators into a single model. Secondly, methodological differences among the included studies may impact our findings. The nature of CBT interventions precludes double‐blinding, and factors such as participants' cognitive levels and therapists' qualifications may influence experimental results, potentially introducing effect biases. These factors may affect the methodological quality and quality assessment of the relevant RCTs. However, we mitigated this limitation by evaluating each selected study according to strict criteria, and the outcome indicator evidence level is moderate. While our moderator analyses aimed to explore heterogeneity across critical covariates, we acknowledge significant methodological constraints when interpreting results from categories containing fewer than five studies. Following methodological best practices, we recognize that moderator analyses with such limited samples lack statistical power and yield potentially unreliable results. For transparency, we here clearly flagged these limitations where such analyses were retained for exploratory purposes only. This methodological constraint necessarily weakens the persuasiveness of findings in these specific moderator categories, and readers should interpret these particular results with appropriate caution. Future meta‐analyses with larger sample sizes across moderator categories are required to validate these exploratory findings. Lastly, the heterogeneity of depressive symptoms and severity among patients may impact our results. CBT is a principle‐based approach, and specific implementation methods may vary across studies, potentially contributing to the heterogeneity of the included studies.

Despite these limitations, our study has significant strengths. While many researchers focus on CBT's efficacy for depression in general, studies specifically examining CBT's impact on suicidal ideation in depressed patients are scarce. Our review addresses this gap by focusing on this specific patient population, providing evidence for optimal CBT therapeutic approaches. Our findings suggest that CBT is an effective treatment for reducing immediate suicidal thoughts in depressed patients, particularly when combined with pharmacological interventions. The optimal therapeutic dose of CBT intervention appears to involve sessions lasting more than 1 h, occurring more than once a week, within a 12‐week intervention period, and utilizing a group intervention format. These findings contribute valuable insights to the field, offering evidence‐based guidance for clinical interventions. However, given the limitations noted, further research is needed to corroborate and expand upon these results, particularly in exploring the mechanisms by which CBT interventions affect suicidal thoughts in patients with depression.

## Ethics Statement

This study has been reviewed and approved by the Ethics Committee of Beijing Normal University. The research protocol was thoroughly evaluated by the University‐level Review Board in accordance with the institution's comprehensive ethical guidelines for research involving human subjects. The study adheres to the principles outlined in the Declaration of Helsinki and complies with all relevant national and international regulations governing research ethics.

## Conflicts of Interest

The authors declare no conflicts of interest.

## Supporting information


**Appendix S1:** Supporting information.

## Data Availability

The dataset(s) supporting the conclusions of this article is(are) included within the article (and its additional file(s)).
