# Supplementary material for: Effectiveness of Cognitive Behavioral Therapy in Reducing Suicidal Ideation and Influential Factors in Patients With Major Depressive Disorder: A Systematic Review and Meta‐Analysis
Source: Psych J. 2025 Jul 3;14(5):734–48. doi: 10.1002/pchj.70034 (PMC12520839; doi:10.1002/pchj.70034)
Supplement: Supplementary file 1 — Appendix S1: Supporting information. [file PCHJ-14-734-s001.docx]

# Database Search Strategy and Results (as of 8 November 2023)

| Database | Search Query Description | Results |
| --- | --- | --- |
| PubMed | #1: Depression OR depressive symptoms OR emotional depression OR Depressive #2: Cognitive Behavioral Therapy OR CBT OR cognitive OR behavioral OR cognitive psychotherapy #3: Suicide OR suicidal behavior OR suicidal ideation #4: Randomized controlled trial OR randomized OR controlled OR trial #5: #1 AND #2 AND #3 AND #4 | 723 |
| Web of Science | #1: Cognitive OR behavior OR cognitive psychotherapy #2: Depression OR depressive symptoms OR emotional depression OR Depressive #3: Suicide OR suicidal behavior OR suicidal ideation #4: Randomized controlled trial OR randomized OR controlled OR trial #5: #1 AND #2 AND #3 AND #4 (excluding Preprint Citation Index) | 2654 |
| Cochrane Library | #1: [Depression] explode all trees #2: depressive symptoms OR emotional depression OR depressive #3: #1 OR #2 #4: [Cognitive Behavioral Therapy] explode all trees #5: CBT OR cognitive OR behavior OR cognitive psychotherapy #6: #4 OR #5 #7: [Suicide] explode all trees #8: Suicidal behavior OR suicidal ideation #9: #7 OR #8 #10: Randomized controlled trial OR randomized OR controlled OR trial #11: #3 AND #6 AND #9 AND #10 | 1035 (11 reviews) |
| EMBASE | #1: Depression (exp) #2: depressive symptoms OR emotional depression OR depressive #3: #1 OR #2 #4: Suicide (exp) #5: Suicidal behavior OR suicidal ideation #6: #4 OR #5 #7: Cognitive Behavioral Therapy (exp) #8: CBT OR cognitive OR behavior OR cognitive psychotherapy #9: #7 OR #8 #10: Randomized controlled trial (exp) #11: randomized OR controlled OR trial #12: #10 OR #11 #13: #3 AND #6 AND #9 AND #12 | 1010 |
